# Supplementary material for: Behavioral and neural responses to social rejection: Individual differences in developmental trajectories across childhood and adolescence
Source: Dev Cogn Neurosci. 2024 Mar 16;66:101365. doi: 10.1016/j.dcn.2024.101365 (PMC10958064; doi:10.1016/j.dcn.2024.101365)
Supplement: Supplementary file 1 — Supplementary material [file mmc1.docx]

# Behavioral and neural responses to social rejection: Individual differences in developmental trajectories across childhood and adolescence

Jeroen D. [Mulder](mailto:https://orcid.org/0000-0002-5553-0856)1, Simone [Dobbelaar](mailto:https://orcid.org/0000-0002-2498-2809)2,3, and Michelle [Achterberg](mailto:https://orcid.org/0000-0001-5874-6495)2,3

1 Department of Methodology and Statistics, Faculty of Social and Behavioral Sciences, Utrecht University

2 Department of Psychology, Education and Child Studies, Erasmus School of Social and Behavioral Sciences, Erasmus University Rotterdam

3 Leiden Consortium Individual Development, Faculty of Social and Behavioral Sciences, Leiden University

**Supplementary Materials**

For readability, the use of the Bayesian multilevel framework for our analyses is discussed only in general terms in the manuscript. Technical details, elaborate explanations, and R code are provided in the online supplementary materials at <https://jeroendmulder.github.io/social-emotion-regulation>

Correspondence concerning the online supplementary materials should be addressed to Jeroen Mulder ([j.d.mulder@uu.nl](mailto:j.d.mulder@uu.nl) )

**Acknowledgements.** Marinus van IJzendoorn, Eveline Crone, and Marian Bakermans-Kranenburg designed the Leiden Consortium on Individual Development (L-CID) experimental cohort-sequential twin study “Samen Uniek” as part of the Consortium on Individual Development (CID). We thank the participating families for their enthusiastic involvement in L-CID. We are grateful to the data-collection and

data-processing team, including all current and former students, research assistants, PhD students, and postdoctoral researchers for their dedicated and invaluable contributions. We also thank Eveline Crone and Stephan Heunis for their help in the preregistration of this study.

**Funding.** This study is part of CID, which was supported by a Gravitation grant awarded by the Dutch Ministry of Education, Culture, & Science, and the Netherlands Organization for Scientific Research [NWO grant number 024.001.003, 2013]. M.A. was funded by the Netherlands Organization for Scientific Research (NWO-VENI).

**Preregistration.** This study was preregistered as Achterberg et al. [(2022).](#_bookmark3)

# Abstract

Dealing with social rejection is challenging, especially during childhood when behavioral and neural responses to social rejection are still developing. In the current longitudinal study, we used a Bayesian multilevel growth curve model to describe individual differences in the development of behavioral and neural responses to social rejection in a large sample (*n >* 500). We found a slight peak in aggression following negative feedback (compared to neutral feedback) during late childhood, as well as individual differences during this developmental phase, possibly suggesting a sensitive window for dealing with social rejection across late childhood. Moreover, we found evidence for individual differences in the linear development of neural responses to social rejection in our three brain regions of interest: The anterior insula, the medial prefrontal cortex, and the dorsolateral prefrontal cortex. In addition to providing insights in the individual trajectories of dealing with social rejection during childhood, this study also makes a meaningful methodological contribution: Our statistical analysis strategy (and online supplementary information) can be used as an example on how to take into account the many complexities of developmental neuroimaging datasets, while still enabling researchers to answer interesting questions about individual-level relationships.

*Keywords:* Bayesian multilevel, growth curves, individual differences in development, social rejection, late childhood

# Behavioral and neural responses to social rejection: Individual differences in developmental trajectories across childhood and adolescence

1. **Introduction**

During the transition from childhood to emerging adolescence (approximately between the ages of 7- to 14-years-old) peer relations and long-lasting friendships become more salient. Dealing with social rejection, that is, regulating one’s emotions and behaviors in social situations, in particular after receiving negative peer feedback, is an important prerequisite for developing and maintaining such relationships. A broad range of literature has shown that receiving negative social feedback can result in reactive aggressive behavior (Dodge et al., [2003;](#_bookmark32) Leary et al., [2006;](#_bookmark50) Nesdale & Lambert, [2007),](#_bookmark57) and that the regulation thereof is related to neural activation (Achterberg et al., [2016;](#_bookmark5) Chester et al., [2014;](#_bookmark20) Riva et al., 2015).

These behavioral and neural responses to social rejection develop across childhood and adolescence (Achterberg et al., [2020;](#_bookmark7) Dobbelaar et al., [2023).](#_bookmark31) However, existing research into development of behavioral aggression has focused largely on group-based averages, obscuring meaningful individual variation across children in development (Chester, [2019).](#_bookmark19) To move towards a more nuanced understanding of behavioral aggression and neurocognitive changes, developmental neuroimaging studies need to characterize individual differences as a variable of interest, as argued by Foulkes and Blakemore [(2018)](#_bookmark36) and Telzer et al. [(2018),](#_bookmark71) amongst others. By addressing individual variability in adolescent development, researchers acknowledge the fact that adolescents, and their brains, develop in meaningfully different ways. This is particularly important when studying behavioral and neural responses to social interactions, as adolescents substantially vary in the quantity and quality of friendships they have, affecting both their behavioral and neural responses to social interactions (Lamblin et al., [2017;](#_bookmark47) Van Harmelen et al., [2017).](#_bookmark77) Some researchers have even proposed that adolescent development is shaped by brain-based individual differences in sensitivity to social contexts, and that individual differences in neurobiology might determine how sensitive an adolescent is to the social context (Schriber & Guyer, [20](#_bookmark66)16). A focus on individual differences in behavioral and neural development also allows for investigating whether such differences are useful predictors for future mental health and well-being (Copeland et al., [2013;](#_bookmark22) Foulkes & Blakemore, [2018;](#_bookmark36) Van Harmelen et al., [2017).](#_bookmark77) Well-being, defined as someone’s appraisal and evaluation of their life (Diener & Ryan, [2009),](#_bookmark29) is a multi-dimensional construct including different facets, such as having impact and purpose, dealing with stress and worry, relationships, self-confidence, and feeling appreciated (Green et al., [2023).](#_bookmark40) Previously, aggression following rejection has been associated with behavioral and peer problems, and a negative spiral of even more peer rejection (Card & Little, [2006;](#_bookmark16) Evans et al., [2021;](#_bookmark33) Lansford et al., [2010),](#_bookmark49) highlighting the important links between aggression regulation and well-being. However, whether individual differences in developmental trajectories are predictive for future well-being is currently unknown.

The current preregistered study investigates individual differences in developmental trajectories of dealing with social rejection (the preregistration is published as Achterberg et al., [2022).](#_bookmark3) Our focus is on behavioral (aggressive) responses, and neural responses to negative social feedback, specifically in three brain regions that have previously been related to the processing of social feedback, namely the anterior insula (AI), the medial prefrontal cortext (MPFC), and the dorsolateral prefrontal cortex (DLPFC). To understand the underlying brain mechanisms, we additionally examine how developmental trajectories of aggression regulation following negative social feedback relate to each other, and to social well-being in early adolescence. To address individual variability in developmental trajectories, we analyze longitudinal behavioral and fMRI data (three waves, measured during childhood and emerging adolescence) in a multilevel modeling framework.

# 1.1 Behavioral and neural responses to social rejection

Regulating behavioral responses following social rejection, operationalized here as aggression regulation following negative social feedback, is essential for children to develop in order to establish and maintain relationships with peers. A recently introduced experimental method for measuring this, which is also used in this study, is the Social Network Aggression Task (SNAT; Achterberg et al., [2016;](#_bookmark5) Achterberg et al., [2018).](#_bookmark6) Using this method, it has been demonstrated that negative social feedback, compared to neutral or positive feedback, can lead to aggression in 7- to 9-year-old children (Achterberg et al., [2018;](#_bookmark6) Achterberg et al., [2017;](#_bookmark8) Dobbelaar et al., [2022),](#_bookmark30) in 9- to 11-year-old children (Achterberg et al., [20](#_bookmark7)20), in typically developing young adults (Achterberg et al., [2016;](#_bookmark5) Van de Groep et al., [2021),](#_bookmark72) and in young adults with a history of antisocial behavior (Van de Groep et al., [2022).](#_bookmark74) By extending the SNAT with fMRI measurements, researchers have investigated relations between social feedback processing and neural (brain) responses, particularly in the AI, MPFC, and DLPFC brain regions. These brain regions are central to several neurodevelopmental models, such as the Social Information Processing Network (SIPN; Nelson et al., [2016;](#_bookmark55) Nelson et al., [2005).](#_bookmark56) This model proposes that social information is processed through communication between three neural systems: A detection node, an affective node (including striatal regions and the anterior insula), and a cognitive regulatory node (including the lateral prefrontal cortex). All three nodes show developmental changes throughout adolescence, and have been related to social information processing within the SNAT design.

It has been shown that both positive and negative social feedback (compared to neutral feedback) in the SNAT result in increased neural activation in the Anterior Cingulate Cortex (ACC) gyrus and bilateral AI (Achterberg et al., [2016;](#_bookmark5) Achterberg et al., [2018;](#_bookmark6) Achterberg et al., [2020;](#_bookmark7) Dobbelaar et al., [2022;](#_bookmark30) Van de Groep et al., [2021).](#_bookmark72) These findings fit with the literature suggesting that the ACC and AI signal for social salience in general (Cheng et al., [2019;](#_bookmark18) Dalgleish et al., [2017;](#_bookmark28) Somerville et al., [2006).](#_bookmark68) Moreover, the social salience networks reported in adults (Achterberg et al., [2016;](#_bookmark5) Van de Groep et al., [2021),](#_bookmark72) middle childhood (Achterberg et al., [2018](#_bookmark6); Dobbelaar et al., [2022)](#_bookmark30) and late childhood (Achterberg et al., [2020)](#_bookmark7) show remarkable resemblances, indicating that *on average* this mechanism is already developed in middle childhood. More importantly, variation in AI activation during negative social feedback has been related to variation in aggression regulation. That is, Achterberg et al. [(2020)](#_bookmark7) previously reported that children with increased activation in the AI during negative social feedback showed more aggression. Interestingly, Chester et al. [(2014)](#_bookmark20) found a similar association, but only in adults with low executive control (and not in adults with high executive control). Possibly, the association between AI activation and behavioral aggression is stronger in childhood than adolescence, as executive control functions increase across development. The current study includes longitudinal measures across childhood and emerging adolescence, such that we can test brain-behavior associations of development in AI activation during social rejection.

Second, the MPFC has been shown to play an important role in social cognition and behavior (Adolphs, [2009;](#_bookmark9) Blakemore, [2008),](#_bookmark11) and is specifically implicated when thinking about others (Apps et al., [2016;](#_bookmark10) Lee & Seo, [2016).](#_bookmark51) Receiving negative social feedback may leave the children wondering what the other might have thought about them (Gallagher & Frith, [2003).](#_bookmark37) Moreover, neurodevelopmental models highlight the MPFC as an important region for the integration of perspectives related to oneself and others (Crone et al., [2020;](#_bookmark23) Crone & Fuligni, [2020),](#_bookmark24) and for sensitivity to social evaluation (Somerville & Casey, [2010),](#_bookmark67) two processes that are especially salient in adolescence. Interestingly, when conducting whole brain analyses, previous studies often failed to find significant neural activation during negative social feedback (Gunther Moor et al., [2010;](#_bookmark41) Guyer et al., [2012)](#_bookmark42) in adolescence. However, studies with a larger sample, and increased statistical power, reported strong activation in the MPFC during social rejection in childhood (Achterberg et al., [2018;](#_bookmark6) Achterberg et al., [2020).](#_bookmark7) As social cognition and behavior are increasingly important during adolescence, activation in this region might show strong development—and strong individual differences in development—during the transition from childhood to adolescence. Developmental differences of the MPFC were recently related to prosocial development in this age period (Van der Meulen et al., [2023),](#_bookmark75) suggesting that individual differences in the development of the MPFC may relate to behavioral development. Previous studies did not reveal associations between aggression regulation following negative social feedback and MPFC activation. However these studies were often underpowered, examined group differences, and/or used aggregated behavioral scores (Chester, [2019).](#_bookmark19)

Third, a brain-behavior association that has been consistently found using the SNAT is the negative association between DLPFC activation during negative social feedback and reactive aggression. That is, consistent with prior experimental studies, increased activation in the DLPFC during social rejection was followed by decreased aggression in adults, suggesting that these individuals were more successful at regulating their behavioral aggression (Achterberg et al., [2016;](#_bookmark5) Riva et al., [2015).](#_bookmark62) Region of interest analyses of the DLPFC in 7- to 9-year-olds provided some indications of an aggression regulation network, but this was not strong enough to be depicted using whole brain-behavior analyses (Achterberg et al., [2018).](#_bookmark6) When examining these same children two years later—now during late childhood—there was a significant association between brain and behavior.

Similar to adults, increased neural activation in the DLPFC was related to decreased behavioral aggression following negative social feedback (Achterberg et al., [2020).](#_bookmark7) Importantly, the children who displayed the largest developmental increases in DLPFC activity across childhood also displayed the largest changes in behavioral aggression. These results suggest that, in addition to being an important region for cool (nonemotional) cognitive control (Crone & Steinbeis, [2017;](#_bookmark25) Luna et al., [2004;](#_bookmark52) Luna et al., [2010)](#_bookmark53) the DLPFC is also important in controlling hot emotional control (Welsh & Peterson, [2014;](#_bookmark79) Zelazo & Carlson, [2012).](#_bookmark81) The current study expands this knowledge by examining functional DLPFC development across a broader age range, including emerging adolescence, and by including both linear and nonlinear development.

# 1.2 Study aims and outline

The aim of this study is threefold. First, we describe developmental trajectories of neural and behavioral (aggressive) responses to social rejection, *allowing for individual differences herein*. That is, we separately describe the individual developmental trajectories of (a) behavioral aggressive responses following social rejection, (b) neural responses in the AI during social rejection, (c) neural responses in the MPFC during social rejection, and (d) neural responses in the DLPFC during social rejection. We focus specifically on the AI, MPFC, and DLPFC brain regions, as these have previously been related to the processing of social rejection. Second, we examine associations between the developmental trajectories of behavioral and neural responses. Third, we test whether individual differences in developmental trajectories of brain and behavior across childhood (7- to 14-year-olds) are predictive for social well-being in (early) adolescence (12- to 15-year-olds).

The paper is organized as follows. In Section 2, we describe our study sample; the measurement instruments that were used; and the statistical analyses, including how missing data, and data nonindependence due to family overlap were controlled for. For readability, the use of the Bayesian multilevel framework for our analyses is discussed only in general terms, and (technical) details, elaborate explanations, and R code are provided in the online supplementary materials at <https://jeroendmulder.github.io/social-emotion-regulation>. Numerical results, organized by study aim, are presented in Section 3. We end with discussion and conclusions in Section 4.

# Methods

- 1. **Participants and procedure**

Participants in this study took part in the longitudinal twin study of the Leiden Consortium on Individual Development (L-CID; Crone et al., [2020).](#_bookmark23) The procedures were approved by the Dutch Central Committee for Human Research (CCMO) and written informed consent was obtained from both parents. Invitations to participate were sent to families with same-sex twins born between 2006 and 2009, within a two-hour radius around the city of Leiden, the Netherlands. Participants were fluent in Dutch and were excluded when they had visual or physical impairments that could disable them from performing the behavioral tasks. The data were collected during annual visits between 2016 and 2021.

Annual visits were either a lab visit (waves 1, 3 and 5), in which families were invited to participate in an fMRI session; or home visits (waves 2, 4 and 6), in which families performed behavioral tasks and completed questionnaires at home (without neuroimaging measures). For the current study, data from the Middle Childhood Cohort collected at the lab visits during waves 1, 3, and 5, and the social well-being questionnaire at wave 6 were used.^[[1]](#footnote-1)^ For details regarding the L-CID study and procedure, see Crone et al. [(2020).](#_bookmark23)

At wave 1 (first fMRI visit, September 2015 to August 2016), 512 children were included (7.02-9.68 years old, *M* = 7*.*94), with 55% being monozygotic. The majority of the sample (91%) was Caucasian and had normal IQ (*M* = 103*.*58, *SD* = 11*.*76), as measured using two subsets of the Wechsler Intelligence Scale for Children, third edition (for details, see Achterberg et al., [2018).](#_bookmark6) Socioeconomic status (based on parental education) was high for 45% of the sample, middle for 46%, and low for 9% of the sample (Crone et al., [2020).](#_bookmark23) 489 children completed the fMRI scan at wave 1. At wave 3 (second fMRI visit, September 2017 to August 2018, 8.98-11.67 years old, *M* = 9*.*98), 456 participants were included, of whom 406 completed the fMRI scan. Wave 5 (third fMRI visit, September 2019 to April 2021, 11.15-14.11 years old, *M* = 12*.*38) included 336 participants, of whom 236 completed the fMRI scan. At wave 6 (June 2021 to October 2021, 11.98-15.10 years old, *M* = 13*.*34), 294 children filled in the digital social well-being questionnaires. Further details about the sample characteristics can be found in Table [**1**](#_bookmark82).

# Measurements

There are three outcomes of interest that were measured for this study: (a) Behavioral aggression following social feedback, measured simultaneously with the fMRI sessions at waves 1, 3, and 5; (b) neural responses in the AI, MPFC, and DLPFC during social feedback, measured at waves 1, 3, and 5; and (c) social well-being, measured at wave 6.

## Behavioral aggression following social feedback

Behavioral aggression following social feedback was measured using the SNAT, which was programmed in Eprime, version 2.0.10.356 (see also Achterberg et al., [2016;](#_bookmark5) Achterberg et al., [2018;](#_bookmark6) Achterberg et al., [2020;](#_bookmark7) Achterberg et al., [2017).](#_bookmark8) One to four weeks prior to the fMRI session, participants filled in a personal profile at home, which was handed in at least one week before the actual fMRI session. The profile page consisted of questions such as: “What is your favorite color?”, “What is your favorite food?”, and “What is your biggest wish?”. Participants were informed that their profiles were reviewed by other, unfamiliar, peers. During the SNAT the participants were presented with pictures and feedback to their personal profile from those unfamiliar peers. Unbeknownst to the participants, others did not judge the profile, and the photos were created by morphing two peers of an existing data base (matching the participants’ age range) into a new, nonexistent peer. Every trial consisted of feedback from a new unfamiliar peer. This feedback could either be positive (visualized by a green thumb up), negative (red thumb down), or neutral (grey circle; see Figure [1, the social feedback event).](#_bookmark86) Peer pictures were randomly coupled to feedback, ensuring equal gender proportions for each type of feedback.

Following each peer feedback, the participants were instructed to send a loud noise blast to this peer (see Figure 1, the noiseblast event). The longer they pressed the button, the more intense the noise would be, which was visually represented by a volume bar. To keep task demands as similar as possible between the conditions, participants were instructed to always press the button, but they could determine the intensity and duration of the noise blast. Participants were instructed to deliver the noise blast by pressing one of the buttons on the button box attached to their legs, with their right index finger. As soon as the participant started the button press, the volume bar started to fill up with a newly colored block appearing every 350 ms. After releasing the button, or at maximum intensity (after 3500 ms), the volume bar stopped increasing and stayed on the screen for the remainder of the 5000 ms. The duration of the button press (in ms) to each negative, neutral, or positive trial was recorded and used as measurement of behavioral aggression in the statistical analyses (see Section 2.3.1). Participants were aware that the peers were not actually receiving the noise blast, but were instructed to respond as if the other peer would receive the noise blast. Although previous experimental tasks have included actual perceived aggression (e.g., using electric shocks; Gianncola & Parot, 2008; for a meta-analyses see Quarmley et al., 2022), we specifically instructed the children to imagine they could retaliate in order to reduce deception, and studies suggest that imagined play also leads to aggression (Konijn et al., [2007).](#_bookmark46)

The SNAT consisted of sixty trials (twenty per condition). An overview of trial order of the SNAT including jitter times is available at [https://osf.io/ycgqe/.](https://osf.io/ycgqe/) Each trial started with a fixation screen (500 ms), followed by the social feedback (2500 ms). After another jittered fixation screen (3000-5000 ms), the noise screen with the volume bar appeared, which was presented for a total of 5000 ms. Before the start of the next trial, another jittered fixation cross was presented (0-11550 ms; Figure [1).](#_bookmark86) The order of trials was semirandomized to ensure that no condition was presented more than three times in a row. The optimal jitter timing and order of events were calculated with Optseq 2 (Dale, [1999).](#_bookmark27) For each wave, the same version of the task was used. In the third fMRI wave we selected different photos of peers, such that they matched the age range of participants.

For the current study, we specifically focused on noise blast duration following negative social feedback, compared to neutral social feedback.

## Neural responses during social feedback

MRI scans were acquired with a Philips Ingenia 3.0 Tesla MR scanner. A standard whole-head coil was used, with foam inserts added to minimize head motion. A screen was placed behind the MRI scanner, such that participants could view the screen displaying the stimuli through a mirror on the head coil. T2*-weighted echo planar imaging (EPI) was used to collect the fMRI scans. The first two volumes were discarded to allow for equilibration of T1 saturation effects (field of view = 220 *×* 220 *×* 111*.*65 mm, TR = 2*.*2 s, TE = 30 ms, FA = 80◦, sequential acquisition, 37 slices, voxel size = 2*.*75 *×* 2*.*75 *×* 2*.*75 mm). A high-resolution 3D T1 scan was collected as anatomical reference (field of view = 224 *×* 177 *×* 168 mm, TR = 9*.*72 ms, TE = 4*.*95 ms, FA = 8◦, 140 slices, voxel size = 0*.*875 *×* 0*.*875 *×* 0*.*875 mm).

fMRI data were analyzed in SPM12 (Wellcome Department of Cognitive Neurology, London). Preprocessing included slice timing correction and correction for rigid body motion. Images were normalized to T1 templates (based on MNI-305 stereotaxic space; Cocosco et al., [1997)](#_bookmark21) using 12-parameter affine transform mapping and nonlinear transformation with cosine basis functions. Volumes of each participant were resampled to 3 *×* 3 *×* 3 mm voxels and were spatially smoothed using a 6 mm full-width -at-half-maximum isotropic Gaussian kernel. Data of participants with at least two blocks of fMRI data with less than 3 mm movement in every direction were included in the analyses (Table [1](#_bookmark82) includes the number of MRI exclusions based on anomalous findings, excessive head motion, or data export/processing errors per wave). Individual participants’ data at each wave were analyzed using a general linear model in SPM12. The onset of feedback delivery was modeled as a zero duration event with positive, neutral and negative feedback added as separate regressors. To model the start of noise blast, the hemodynamic response function (HRF) was modeled for the length of the noise blast duration. Noise blasts following positive, neutral, and negative feedback were modeled as separate regressors (Achterberg et al., [2018).](#_bookmark6) This study focuses specifically on neural responses during the social feedback event. Longitudinal trajectories of the noise blast event are described in Dobbelaar et al. [(2023).](#_bookmark31) Trials on which participants did not respond in time were marked invalid and excluded from further analyses. Six motion regressors were added as covariates of no interest. Least-squares parameter estimates of height of the best fitting canonical HRF for each condition were used in pairwise contrasts. The focus of this study was on the contrast negative versus neutral feedback.

Based on previous findings in an adult sample (*N* = 30, 18–30 years old) by Achterberg et al. [(2016),](#_bookmark5) the AI, MPFC, and right DLPFC were selected as regions of interest (ROI, see Figure [2).](#_bookmark87) Specifically, we selected the bilateral AI region (414 voxels) based on the left and right insular cortex clusters of the conjunction contrast (see supplementary Table S3 of Achterberg et al., [2016,](#_bookmark5) contrast conjunction of “positive *>* neutral” and “negative *>* neutral”, Cluster L Insular Cortex and R Insular Cortex). The MPFC region (379 voxels) was extracted from the contrast “negative *>* neutral” feedback (see Supplementary Table S2 of Achterberg et al., [2016,](#_bookmark5) Cluster L Frontal Pole). The right DLPFC region (1144 voxels) was extracted from the whole brain-behavior regression analyses (see supplementary Table S4 of Achterberg et al., [2016](#_bookmark5), contrast “negative *>* neutral feedback” with noise blast duration difference score as negative regressor, Cluster R Middle Frontal Gyrus). Parameter estimates were extracted using the MarsBar toolbox (Brett et al., [2002)](#_bookmark14) for the contrast “negative feedback *>* neutral feedback”, which was used as a measure of neural activity during social rejection. These fMRI brain data analyses resulted in individual- and wave-specific contrast scores per ROI, representing the mean difference in brain activity between the negative and neutral social feedback conditions.

## Social well-being questionnaire

The social well-being questionnaire was filled in by participants at wave 6 and consisted of 35 items. A complete overview of the questionnaire including all items and response categories is available at [https://osf.io/fseq8/.](https://osf.io/fseq8/) It was constructed from five subscales: Ten items from the Adolescent Wellbeing Paradigm (AWP; Green et al., [2023),](#_bookmark40) ten items from the World Health Organization Quality of Life Scale (WHOQoL; Vahedi, [2010),](#_bookmark73) and three subscales (each five items) from the Harter’s Self-Perception Profile for Adolescents (SPPA; Harter, [1988;](#_bookmark43) Wichstraum, [1995),](#_bookmark80) specifically the subscales Social Competence (SC), Close Friendships (CF) and Global Self-worth (GS). All items were answered on a four-point Likert scale, with low scores indicating low social well-being and high scores indicating high social well-being. Instructions in each of the subscale manuals were followed for the handling of missing data and scoring of subscale scores, resulting in simple mean scores per subscale.

# Statistical analyses

In this section, the statistical analyses are described in general terms. For Aim 1—describing development in behavioral and neural responses to social rejection, and individual differences herein—brain and behavioral data were analyzed with growth curve models in a Bayesian multilevel modeling framework. For Aims 2 and 3—investigating the relationships between individual development in behavioral responses, individual development in neural responses, and later social well-being—a structural equation modeling (SEM) approach was used. Technical details on these analyses (e.g., model equations, the fitting procedure, assessment of convergence, and model fit), R code, and a rationale for the modeling decisions that were made, can be found in this study’s online supplementary materials.

## Growth curve models in a Bayesian multilevel framework (Aim 1)

To describe developmental trajectories of (aggressive) behavioral and brain responses, growth curve models were fitted for each outcome in a Bayesian multilevel framework. The multilevel framework was used to allow for individual differences in the development of brain and behavioral responses, and to more easily accommodate the individual variation in age at each measurement wave (i.e., there is substantial variability in participants’ age at each measurement occasion, see Section 2.1). The Bayesian framework was used because it is more flexible in accommodating some characteristics of the data, such as dropout of participants across time, censoring of the behavioral response data at 3500 (ms), and potential nonnormality. The models were fitted using the package brms (version 2.18.0; Bürkner, [2017)](#_bookmark15) in R (version 4.2.2; R Core Team, [2022).](#_bookmark61) Analysis of the behavioral response data is discussed first. The data have a four-level structure, with the sixty repeated trials nested within three measurement waves, nested within individuals, nested within families. Using a multilevel model, we can estimate individual behavioral responses following social rejection at the trial level (level 1), model the development in these responses across a participant’s age at the wave level (level 2), describe individual differences within families in this development at the individual level (level 3), and account for twin-dependence in the measurements at the family level (level 4). It is important to note that because our data is twin data, individual differences here are a combination of differences between individuals *within a given family/twin-pair* (level 3) and differences *between such families/twin-pairs* (level 4). For the current study, this differentiation is not of substantive interest, and is only made to control for the nonindependence of observations in our statistical analyses. Intercept-only models were run first to assess the proportion of observed variance that can be explained by each of the levels, as expressed in an intraclass-correlation coefficient (ICC; Gelman & Hill, [2007).](#_bookmark38)

From a multilevel model we can extract various components relevant for Aim 1. The model’s *fixed effect* (FE) parameters capture *average* change, that is, averaging across individuals within families (level 3) and across families (level 4), does an individual’s behavioral response following social rejection change as the individual’s age increases? Because behavioral change across time is hardly ever linear, we include FE parameters for both linear and quadratic changes across time. In total, three FE parameters from the model are of interest: An intercept, which captures the expected behavioral response to negative feedback (compared to the neutral condition) at the mean age (approximately nine years and nine months)^[[2]](#footnote-2)^, an expected linear slope in behavioral response at mean age, and an expected quadratic slope in behavioral response at mean age. From hereon we jointly refer to the intercept and slopes as *growth components*. These results are presented in Table [2,](#_bookmark83) and discussed in the results Section.

The multilevel model contains random effect (RE) terms for the growth components at the individual level and the family level. The inclusion of these terms in the model implies that the estimated development (as captured by the growth components) can vary from individual-to-individual within a family (i.e., through the RE terms at level 3), and between families (i.e., through the RE terms at level 4). Standard deviations of the RE terms are then measures of across adolescent (but within family) and between-family variability in the development of behavioral and neural responses, respectively. By extracting RE terms for each individual and family, we can create individual-specific growth components. These components serve as input for the second-part of the data analysis (see Section 2.3.2).

The analysis procedure of the neural responses was largely similar to the analysis procedure for the behavioral responses: For each ROI, growth curve models were fitted in a Bayesian multilevel framework, and FEs (averaged across individuals and families) and REs (both individual- and family-specific) of the growth components were extracted herefrom. There was one notable exception. As described in Section 2.2.2, preprocessing of the fMRI data resulted in contrast score averages across trials rather than trial-specific scores. Hence, for the fMRI data, **the neural responses during social rejection** (compared to the neutral condition) do not have to be estimated anymore as part of the multilevel model.

Therefore, for the fMRI data, a three-level multilevel model was used in which the trial level was omitted.

The Bayesian framework was used to handle multiple complicating factors of the data. First, it accommodated censoring in the behavioral data (at 3500 ms) by integrating censored values out. Second, to prevent unnecessary loss of data, missing data for the outcomes were imputed as part of the model fitting procedure under the assumption of missing at random. Third, because the data showed increased kurtosis, a Student *t* distribution was used for the outcome to increase model fit (compared to assuming a

Gausian-distributed outcome). Ultimately, a Bayesian fitting procedure does not result in a single point estimate of the model parameters, but rather in a distribution of likely values for each parameter (i.e., the posterior distribution). We specified the Bayesian fitting procedure such that it resulted in a thousand sets of plausible values for individual-specific growth components for each outcome. These data sets were used as input for the structural equation model for investigating Aims 2 and 3.

## Structural equation model (Aims 2 and 3)

Structural equation modeling was used to investigate the associations between the individual-specific growth components of the behavioral and neural responses (Aim 2), and between later social well-being (Aim 3). First, a one-factor confirmatory factor analysis was performed on the five social well-being subscale means. If the one-factor model for social well-being showed good model fit, we would use the growth components to predict a common social well-being factor. If the one-factor model showed bad model fit, the growth components would predict each of the social well-being subscales separately.

Second, a multivariate regression model was specified with a latent social well-being factor (or the subscales separately) as the outcome(s), and the estimated growth components from the Bayesian multilevel model as predictors. In this model, the predictors were allowed to covary freely with each other such that associations between development in behavioral responses and development in neural activation could be estimated (Aim 2). The regression coefficients represent the relationship between development in behavioral and neural responses to social rejection and social well-being (subscales) in adolescence (Aim 3). The models were fitted using the R package lavaan (version 0.6.16; Rosseel, [2012).](#_bookmark63)^[[3]](#footnote-3)^

As explained in Section 2.3.1, a thousand sets of plausible values for the growth components were extracted from the Bayesian multilevel model. Hence, the multivariate regression model was fitted a thousand times, once for each set of plausible values. This was done using the R package semTools (version 0.5.6; Jorgensen et al., [2022).](#_bookmark45) Parameter estimates of the thousand fitted SEM models were averaged to create a single point estimate of the associations amongst the growth components, and their relation with later social well-being (either a single common social well-being factor, or its five separate subscales). Standard errors for these parameters were pooled following the rules by Rubin [(1987).](#_bookmark64)

# Results

In this section we only highlight model results that are directly related to this study’s Aims. The full set of numerical results can be found in the online supplementary materials.

# Individual differences in development of neural and behavioral responses (Aim 1)

For our first aim, Bayesian multilevel growth curve models were fitted to the behavioral and neural data. Table [2](#_bookmark83) contains the ICCs for the behavioral and neural outcomes across the grouping levels. For the behavioral data, approximately 29% of the observed variance can be explained by variation across measurement waves; which we attempt to explain as a function of participants’ age in the growth curve models. Only small amounts of variance can be explained by the individual- and family-levels for both the behavioral and neural outcomes. Nevertheless, Bayesian hypothesis tests indicate that the posterior probability of the ICCs being greater than zero exceeds 95% for all outcomes, and therefore we decided to control for individual- and family-dependencies in the data (i.e., through inclusion of an individual- and family-level in the multilevel models).

Table [3](#_bookmark84) contains the 95% credible intervals for the FEs of the growth components, and standard deviations of the REs (at both the individual- and family-level) of the growth components. Results for the FEs are also visualized in Figure [3,](#_bookmark88) which shows the model-predicted development across adolescence for behavioral aggressive response following negative versus neutral social feedback (Figure [3a),](#_bookmark88) and the neural responses during negative versus neutral social feedback in the AI (Figure [3b),](#_bookmark88) MPFC (Figure [3c),](#_bookmark88) and DLPFC (Figure [3d).](#_bookmark88)

For behavioral aggression, results show that there is 95% certainty that the expected behavioral response at the mean age (approximately nine years and nine months) lies between 1.31 and 1.49 seconds. The REs imply that there is evidence of differences between individuals (within families) herein—with the standard deviation of the RE at the individual level estimated to be between 0.03 and 0.42—as well as differences between families—with the standard deviations of the REs at the family level estimated to be between 0.15 and 0.43. Linear development of behavioral aggression at the mean age is estimated to lie between -0.05 and 0.02, with the standard deviation of the RE estimated to lie between 0.00 and 0.11 for the individual level, and between 0.01 and 0.16 at the family level. This implies that there is no, to little evidence of differences between individuals and families in linear development, respectively. Quadratic development at the mean age is estimated to be slightly negative, lying between -0.06 and -0.03. This implies that expected development herein follows an inverted-U shape, with behavioral aggression following negative feedback peaking in late childhood, and decreasing thereafter. There is no evidence of between-individual or between-family differences herein.

For neural responses in the AI, the fixed effects show that there is 95% certainty that expected response at the mean age lies between 0.42 and 1.03. The random effects imply that there is evidence of between-individual (within-families), and between-family differences herein. Linear development of AI response at the mean age is estimated to lie between -0.29 and -0.02, with results indicating some evidence of differences between individuals and between families herein. Quadratic development at the mean age is estimated to lie between -0.01 and 0.13, implying that there is no evidence of a quadratic trend in AI development across adolescence. Additionally, there is no evidence of differences between individuals or between families herein. Thus, in general we found evidence for increased AI activity during social rejection, and a linear decrease herein (but no quadratic development). Furthermore, results also show individual differences in linear development.

For neural responses in the MPFC, the results for the fixed effects show that there is 95% certainty that expected response at mean age lies between 0.52 and 1.20. The random effects imply that there are significant differences between individuals (within families) herein, as well as significant between-family differences. Linear development of MPFC at mean age is estimated to lie between -0.17 and 0.10, with only marginal evidence of differences between individuals in linear development, and no evidence of differences between families. Quadratic development at mean age is estimated to lie between -0.01 and 0.13. Results show no evidence of differences between individuals herein. Thus, in general we found evidence for increased **MPFC activity during social rejection**, but no overall (linear or quadratic) development herein. However, results do show individual differences in linear development (i.e., for some individuals there is a positive linear development, and for some a negative).

Finally, for neural responses in the DLPFC, results for the fixed effects show that there is 95% certainty that expected response at mean age lies between -0.65 and -0.09. The random effects provide only marginal evidence that are between individual-, and between family differences families differences herein. Linear development at mean age is estimated to lie between -0.14 and 0.11, with again only marginal evidence of between individual and between family differences. The results show no evidence for a significant quadratic trend on average for the development of DLPFC responses, and do not suggest differences between individuals or between families. Thus, in general we found evidence for decreased DLPFC activity during social rejection, but no overall (linear or quadratic) development herein. However, results do show individual differences in linear development.

# Associations between growth components of behavioral and neural responses (Aim 2)

For Aims 2 and 3, a single multivariate regression model was fitted in which the growth components predicted later social well-being (of interest for Aim 3), and the predictors freely covaried with each other (of interest for Aim 2). In total, 66 covariances between the individual-level growth components of neural and behavioral responses to social feedback were estimated. Of these, two covariances were significant at the *α < .*05 level. The covariance between the expected AI response at mean age (intercept) and the linear development in AI response at mean age (linear slope) was estimated to be -0.123, *SE* = 0*.*054, *t*(978*.*689) = *−*2*.*280, *p* = *.*023. This implies that individuals with a higher AI response at mean age tend to have a steeper linear decrease in AI response. Furthermore, the covariance between the expected MPFC response at mean age (intercept) and the quadratic slope of MPFC at mean age was estimated to be *−*0*.*111, *SE* = 0*.*051, *t*(1012*.*368) = *−*2*.*194, *p* = *.*028. This implies that individuals with a higher expected MPFC at mean age also show a less curvilinear (i.e., more linear) development. So overall, two significant associations of the estimated individual trajectories of AI and MPFC were found. However, the hypothesized covariances between behavioral and neural responses to social feedback did not reach significance.

# Prediction of social well-being subscales (Aim 3)

The exact specification of social well-being in the multivariate regression model was determined based on how well the social well-being subscales could be represented as a unidimensional construct. To this end, a one-factor confirmatory factor analysis model was fitted to the five subscale measures. Estimates of the factor loadings *λ*, unique subscale variances *θ*, and the common social well-being factor variance *ψ* are reported in Table [4.](#_bookmark85) A unidimensional structure for the social well-being subscales showed substantial misfit to the data, *χ*^2^(5) = 49*.*269, *p < .*001, *CFI* = *.*922, *T LI* = *.*844, *RMSEA* = 0*.*178. Therefore, subscales were included as separate outcomes in the multivariate regression model rather than a common social well-being factor. Such a model, in which the exogenous predictors freely covary amongst each other, and in which all outcome residuals freely covary amongst each other, is saturated, implying perfect fit.

In this model, none of the growth components significantly predicted any of the social well-being subscales. Hence, we did not find the hypothesized significant covariances between individual developmental trajectories of behavioral and neural responses to social feedback across childhood and adolescence, and the social well-being subscales measured in early adolescence.

# Discussion

The regulation of negative emotions during social interaction is an essential quality for developing and maintaining social relations, and there are many individual differences in how children respond to social rejection. Although prior literature has linked social development to changes in behavioral (aggressive) responses and neural activation, previous literature has mostly focused on group-based aggregates, limiting our knowledge on individual differences in development (Chester, [2019).](#_bookmark19) Complementing existing research, this preregistered study focuses on the development of behavioral aggression and neural responses in the AI, MPFC, and DLPFC during social rejection, and places individual differences in such development front and center. The renewed focus on individual variability endorses the fact that adolescents’ behavioral and neural responses to social interaction develops in meaningfully different ways (Foulkes & Blakemore, [2018;](#_bookmark36) Telzer et al., [2018),](#_bookmark71) and allows for investigating if such individual differences are predictive of, for example, future health outcomes (Copeland et al., [2013;](#_bookmark22) Foulkes & Blakemore, [2018;](#_bookmark36) Van Harmelen et al., [2017).](#_bookmark77) In this study, we made use of data of L-CID (Crone et al., [2020),](#_bookmark23) which is a longitudinal (experimental) data set containing neural (fMRI) and behavioral measurements following social interaction (for more information, see [https://www.developmentmatters.nl/)](https://www.developmentmatters.nl/). To describe linear and quadratic development of behavioral and neural responses, as well as individual differences herein (Aim 1), we fitted Bayesian multilevel growth curve models. Results from the multilevel models served as input for a structural equation model, in which we simultaneously investigated intercept-slope associations among brain and behavioral development (Aim 2), and whether or not individual behavioral and neural development could predict social well-being (Aim 3).

The main findings of this study are threefold: First, average behavioral development was found to be nonlinear (quadratic), with a peak in behavioral response during late childhood. Individual differences were found primarily in the intercept (expected behavioral response at mean age) and to a lesser degree in the linear slope. Secondly, in line with our expectations, we found individual differences in the linear development of **neural responses during social rejection**. Third, we did not find associations between the estimated individual trajectories of brain and behavioral response, nor were these estimated individual trajectories predictive for future self-reported social well-being. Below, we discuss the theoretical and methodological implications of these main findings further.

# Late childhood as sensitive window for social development

We found that behavioral response following social rejection (as measured by aggression following negative versus neutral feedback) peaks during late childhood. The REs in the multilevel models described general linear and quadratic development at the mean age of approximately nine years and nine months. Based on the estimated standard deviations of the REs, we found evidence for individual differences in the intercept (i.e., expected behavioral response at mean age). Note that here, individual differences are a combination of both differences within- families at the individual level, and between-families at the family level. Furthermore, there was some evidence for individual differences in linear slope between families, but these effects were less pronounced. This suggests that children may differ in their response to rejection in late childhood, but that the developmental trajectories (i.e., a peak in aggression in late childhood) are relatively similar between children. Although most prior developmental studies have focused on adolescent specific peaks in social behavior (cf. Brechwald & Prinstein, [2011;](#_bookmark13) Casey et al., [2010;](#_bookmark17) Somerville & Casey, [2010;](#_bookmark67) Steinberg, [2008;](#_bookmark69) Steinberg & Morris, [2001),](#_bookmark70) our results suggest that late childhood is also an important period for social development, specifically for dealing with social rejection. Prior work on reactive aggression also reported a peak in late childhood (Cui et al., [2016),](#_bookmark26) with decreases in aggression towards adolescence (Fite et al., [2008).](#_bookmark35) This peak in aggression in late childhood may be explained by delayed development of inhibition of aggression following negative feedback, compared to inhibition of aggression following neutral feedback (Dobbelaar et al., [2023).](#_bookmark31) However, although social rejection is a challenging experience for all children, there are pronounced differences in how children deal with such rejection. While some socially rejected children suffer from widespread and persistent impairments in mental health (i.e., internalizing and externalizing problems; Ladd, [2006;](#_bookmark48) Prinstein & Aikins, [2004;](#_bookmark59) Prinstein & La Greca, [2004),](#_bookmark60) other children seem more resilient in dealing with social rejection (Ioannidis et al., [2020;](#_bookmark44) Van Harmelen et al., [2021).](#_bookmark76) Until now there was little insight on where in the developmental process these individual differences emerge. Our findings add to the existing literature by providing evidence for individual differences during late childhood. Possibly, the peak in aggression following negative feedback during late childhood, and individual differences herein, suggests an undiscovered sensitive period in development. This sensitive window might provide a window of opportunity for interventions that foster social development in youth.

# Individual differences in the linear development of neural responses during social rejection

With regards to overall development of neural responses, the results provide evidence of a negative linear development in the AI. This implies that, in general, the AI response during social rejection is expected to decrease between ages nine and ten, leveling off again in emerging adolescence. Additionally, in line with earlier empirical and theoretical studies, we report evidence for individual differences in linear development of all ROIs (Bottenhorn et al., [2023;](#_bookmark12) Foulkes & Blakemore, [2018).](#_bookmark36) Furthermore, neural sensitivity to social feedback may be shaped by social experiences (Rudolph et al., [2021),](#_bookmark65) that can substantially differ between individuals. However, very few studies have investigated brain development across childhood. The main reason for this is that scanning children is more challenging than scanning adolescents or adults (Achterberg & Van der Meulen, [2019;](#_bookmark4) O’Shaughnessy et al., [2008).](#_bookmark58) Nevertheless, our findings indicate that there is evidence of individual differences in brain development during childhood, and highlight that future studies should also include participants below the age of twelve. Notably, we did not find evidence of quadratic trends in the developmental trajectories, nor in general, nor at an individual level. Prior studies have suggested nonlinear development across puberty and adolescence and our results add to this literature by showing that functional brain development across childhood seems mostly linear (Gracia-Tabuenca et al., [2021;](#_bookmark39) Vijayakumar et al., [201](#_bookmark78)9).

# Testing brain-behavior associations: Methodological considerations

We did not find evidence for associations between the estimated growth components of behavioral and neural responses themselves (Aim 2), nor were we able to predict future social well-being from the individual growth components (Aim 3). That is, our analyses did not provide any evidence that individual differences in development are meaningfully related to each other, or to future social well-being. This stands in contrast to previous studies based on (parts of) the same data (cf. Achterberg et al., [2016;](#_bookmark5) Achterberg et al., [2020;](#_bookmark7) Dobbelaar et al., [2022;](#_bookmark30) Dobbelaar et al., [2023;](#_bookmark31) Van de Groep et al., [2021).](#_bookmark72) For example, it was found that behavioral aggression regulation across time was associated with DLPFC activation across time (Achterberg et al., [2020).](#_bookmark7)

There are a couple of potential explanations for this seeming discrepancy. First, this research project is ambitious in its scope, and utilized a complex study design (e.g., longitudinal twin data, in which individuals inevitably drop out, and in the presence of censoring). Our specific setup therefore requires a large number of individuals and repeated measures in order to achieve adequate statistical power. While this study is amongst the first in the literature to attempt to collect repeated MRI data in children at this scale, the sample size might still be too small to detect the many, and arguably small neural relationships that are targeted here (Marek et al., [2022).](#_bookmark54) Second, the statistical analyses in this study deviate in some important ways from previous studies into this topic. The deviations concern the handling of missing data, censoring in the data, and individually-varying times of observations of participants. Such methodological and statistical differences between studies can lead to differences in results, and consequently differences in conclusions that are drawn. This underlines the importance of making informed decisions about the methodological and statistical choices that researchers have a priori, and recording these in a preregistration, or even better, a registered report, with the inclusion of extensive peer reviewing. It is also important to engage in team science, with interdisciplinary collaborations on research projects to get different perspectives on the subject-matter and analysis strategy (Fair et al., [2021).](#_bookmark34)

# Limitations and future directions

This is the first study to describe individual developmental trajectories of behavioral and neural responses to social rejection using a Bayesian multilevel modeling framework. Our statistical analysis strategy makes a meaningful methodological contribution by showing how to take into account the many complexities of developmental neuroimaging datasets, whilst still being able to acknowledge and describe individual variation in development. Despite these strengths, there are several limitations that may have contributed to the null results of this specific study, and which should be taken into account for future research.

First, the aggression measure of the SNAT reflects *hypothetical* aggression or frustration, as participants were aware that the peers did not actually receive the noise blast. This decision was based on previous studies using a similar design (Konijn et al., [2007),](#_bookmark46) but future studies may separate real aggression (that occurs naturally, in daily life) from hypothetical aggression to test the neural differences in these two types of aggression.

Second, some of the prior studies using the SNAT paradigm relied on the same sample: Achterberg et al. [(2018)](#_bookmark6), Achterberg et al. [(2020),](#_bookmark7) Dobbelaar et al. [(2022),](#_bookmark30) Dobbelaar et al. [(2023),](#_bookmark31) and the current study all rely on the L-CID Middle Childhood Cohort (MCC) sample. Although the study aims and research questions investigated in each of these studies are distinct, and findings were based on (a combination) of different measurement waves, future studies should aim to examine behavioral and neural responses to social rejection using the SNAT in various different samples. Currently the SNAT is already being adapted and included in several international and interdisciplinary collaborations. An important and interesting future direction is to examine overlapping and distinct results of the SNAT using meta-analytical techniques.

Third, while the multilevel model for behavioral outcomes contained a trial-level in which behavioral response following social rejection was estimated, for neural outcomes, such a trial-level was omitted: Neural responses (contrasts) were computed in the preprocessing step of the MRI data and then included as observed variables in the multilevel models for neural outcomes. The disadvantage of this two-step procedure is that the uncertainty of estimating neural responses in the preprocessing step is not carried over to the multilevel model. Consequently, credible intervals for parameters from the multilevel model might be (slightly) too narrow. Future studies are recommended to setup their preprocessing pipeline for MRI data in such a way that trial-level MRI measurements can be analyzed, thereby more accurately incorporating the fact that neural responses during social feedback are estimates rather than observed values.

Fourth, a potential explanation for our lack of significant relations between the individual level growth-components and later social-wellbeing might be the time interval between the well-being measurements, and the age to which the growth components relate. These growth components capture linear and quadratic growth at 9 years and 9 months, and it is possible that three and a halve years (the approximate average number of years between 9 years and 9 months, and the average age at which social wellbeing was measured) is too long (or short) a time window for there to be relationships between neural and behavioral response development, and future well-being. There is little to no literature to guide the optimal selection of a time lag between the age at which we capture development, and the age at which we measure well-being. For the design of future studies, theories of social feedback processing need to be extended in order to make informed decisions about how to best investigate this.

Finally, a next step would be to investigate *what* drives the individual differences in the development of social rejection responses. A potential source of such individual differences is puberty, as studies have shown that the timing of pubertal onset varies substantially, and is influenced by both genetic and environmental factors (Zhu, Kusa & Chan, 2018). Therefore, the inclusion of pubertal measures (both self-reported as well as hormonal data from saliva and/or hair) as potential drivers of individual differences in analyses regarding behavior development and brain maturation is an interesting avenue for future research.

# Conclusion

Dealing with social rejection, or negative peer feedback, can be challenging, specifically for children as their social and emotional regulation skills are still developing. Prior research has focused largely on group-based averages of this development, obscuring meaningful individual variation in development. Here, we employed a Bayesian multilevel modeling framework to describe individual differences in the development of behavioral and neural responses to negative social feedback. We found a peak in behavioral reactivity to social rejection across late childhood, as well as individual differences during this developmental phase. Moreover, we report evidence for individual differences in the linear development of neural responses during social rejection in our three brain regions of interest: the AI, MPFC and DLPFC. Our follow-up analyses did not provide evidence for associations between individual trajectories of brain and behavior, or later social well-being. In addition to providing insights in the individual trajectories of behavioral and neural responses to social rejection during childhood, this study also makes a meaningful methodological contribution. That is, our statistical analysis strategy can be used as an example of how to take into account the many complexities of developmental neuroimaging datasets, while still enabling researchers to answer interesting questions about individual-level relationships.

# References

Achterberg, M., Mulder, J. D., Dobbelaar, S., Heunis, S., & Crone, E. (2022). *Individual differences in developmental trajectories of social emotion regulation from childhood to emerging adolescence* (Preregistration) [Publisher: Open Science Framework]. Retrieved August 10, 2023, from <https://doi.org/10.17605/OSF.IO/HDRZC>

Achterberg, M., & Van der Meulen, M. (2019). Genetic and environmental influences on MRI scan quantity and quality. *Developmental Cognitive Neuroscience*, *38*, 100667. <https://doi.org/10.1016/j.dcn.2019.100667>

Achterberg, M., Van Duijvenvoorde, A. C. K., Bakermans-Kranenburg, M. J., & Crone, E. A. (2016). Control your anger! The neural basis of aggression regulation in response to negative social feedback. *Social Cognitive and Affective Neuroscience*, *11* (5), 712–720. <https://doi.org/10.1093/scan/nsv154>

Achterberg, M., Van Duijvenvoorde, A. C. K., Van Der Meulen, M., Bakermans-Kranenburg, M. J., & Crone, E. A. (2018). Heritability of aggression following social evaluation in middle childhood: An fMRI study. *Human Brain* *Mapping*, *39* (7), 2828–2841. <https://doi.org/10.1002/hbm.24043>

Achterberg, M., Van Duijvenvoorde, A. C. K., Van IJzendoorn, M. H., Bakermans-Kranenburg, M. J., & Crone, E. A. (2020). Longitudinal changes in DLPFC activation during childhood are related to decreased aggression following social rejection. *Proceedings of the National Academy of Sciences*, *117* (15), 8602–8610. <https://doi.org/10.1073/pnas.1915124117>

Achterberg, M., Van Duijvenvoorde, A. C., Van Der Meulen, M., Euser, S., Bakermans-Kranenburg, M. J., & Crone, E. A. (2017). The neural and behavioral correlates of social evaluation in childhood. *Developmental Cognitive Neuroscience*, *24*, 107–117. <https://doi.org/10.1016/j.dcn.2017.02.007>

Adolphs, R. (2009). The social brain: Neural basis of social knowledge. *Annual Review of Psychology*, *60* (1), 693–716. <https://doi.org/10.1146/annurev.psych.60.110707.163514>

Apps, M. A., Rushworth, M. F., & Chang, S. W. (2016). The anterior cingulate gyrus and social cognition: Tracking the motivation of others. *Neuron*, *90* (4), 692–707. <https://doi.org/10.1016/j.neuron.2016.04.018>

Blakemore, S.-J. (2008). The social brain in adolescence. *Nature Reviews Neuroscience*, *9* (4), 267–277. <https://doi.org/10.1038/nrn2353>

Bottenhorn, K. L., Cardenas-Iniguez, C., Mills, K. L., Laird, A. R., & Herting, M. M. (2023). Profiling intra- and inter-individual differences in brain development across early adolescence. *NeuroImage*, *279*, 120287. <https://doi.org/10.1016/j.neuroimage.2023.120287>

Brechwald, W. A., & Prinstein, M. J. (2011). Beyond homophily: A decade of advances in understanding peer influence processes. *Journal of Research on Adolescence*, *21* (1), 166–179. <https://doi.org/10.1111/j.1532-7795.2010.00721.x>

Brett, M., Anton, J. L., Valabregue, R., & Poline, J.-B. (2002). Region of interest analysis using an SPM toolbox. *NeuroImage*, *16*, 372–373. <https://doi.org/10.1016/S1053-8119(02)90013-3>

Bürkner, P.-C. (2017). Brms: An R package for Bayesian multilevel models using Stan. *Journal of Statistical Software*, *80* (1). <https://doi.org/10.18637/jss.v080.i01>

Card, N. A., & Little, T. D. (2006). Proactive and reactive aggression in childhood and adolescence: A meta-analysis of differential relations with psychosocial adjustment. *International Journal of Behavioral Development*, *30* (5), 466–480. <https://doi.org/10.1177/0165025406071904>

Casey, B., Jones, R. M., Levita, L., Libby, V., Pattwell, S. S., Ruberry, E. J., Soliman, F., & Somerville, L. H. (2010). The storm and stress of adolescence: Insights from human imaging and mouse genetics. *Developmental Psychobiology*, n/a–n/a. <https://doi.org/10.1002/dev.20447>

Cheng, T. W., Vijayakumar, N., Flournoy, J. C., Op De Macks, Z., Peake, S. J., Flannery, J. E., Mobasser, A., Alberti, S. L., Fisher, P. A., & Pfeifer, J. H. (2019). *Feeling left out or just surprised? Neural correlates of social exclusion and over-inclusion in adolescence* (preprint). Neuroscience. <https://doi.org/10.1101/524934>

Chester, D. S. (2019). Beyond the aggregate score: Using multilevel modeling to examine trajectories of laboratory-measured aggression. *Aggressive Behavior*, *45* (5), 498–506. <https://doi.org/10.1002/ab.21837>

Chester, D. S., Eisenberger, N. I., Pond, R. S., Richman, S. B., Bushman, B. J., & DeWall, C. N. (2014). The interactive effect of social pain and executive functioning on aggression: An fMRI experiment. *Social Cognitive and Affective Neuroscience*, *9* (5), 699–704. <https://doi.org/10.1093/scan/nst038>

Cocosco, C. A., Kollokian, V., Kwam, R. K.-S., & Evans, A. C. (1997). BrainWeb: Online Interface to a 3D MRI Simulated Brain Database. *Proceedings of 3rd International* *Conference on Functional Mapping of the Human Brain*, *5*.

Copeland, W. E., Wolke, D., Angold, A., & Costello, E. J. (2013). Adult psychiatric outcomes of bullying and being bullied by peers in childhood and adolescence. *JAMA Psychiatry*, *70* (4), 419. <https://doi.org/10.1001/jamapsychiatry.2013.504>

Crone, E. A., Achterberg, M., Dobbelaar, S., Euser, S., Van Den Bulk, B., Der Meulen, M. V., Van Drunen, L., Wierenga, L. M., Bakermans-Kranenburg, M. J., & Van IJzendoorn, M. H. (2020). Neural and behavioral signatures of social evaluation and adaptation in childhood and adolescence: The Leiden consortium on individual development (L-CID). *Developmental Cognitive Neuroscience*, *45*, 100805. <https://doi.org/10.1016/j.dcn.2020.100805>

Crone, E. A., & Fuligni, A. J. (2020). Self and others in adolescence. *Annual Review of* *Psychology*, *71* (1), 447–469. <https://doi.org/10.1146/annurev-psych-010419-050937>

Crone, E. A., & Steinbeis, N. (2017). Neural perspectives on cognitive control development during childhood and adolescence. *Trends in Cognitive Sciences*, *21* (3), 205–215. <https://doi.org/10.1016/j.tics.2017.01.003>

Cui, L., Colasante, T., Malti, T., Ribeaud, D., & Eisner, M. P. (2016). Dual trajectories of reactive and proactive aggression from mid-childhood to early adolescence: Relations to sensation seeking, risk taking, and moral reasoning. *Journal of Abnormal Child* *Psychology*, *44* (4), 663–675. <https://doi.org/10.1007/s10802-015-0079-7>

Dale, A. M. (1999). Optimal experimental design for event-related fMRI. *Human Brain Mapping*, *8* (2-3), 109–114. https://doi.org/10.1002/(SICI)1097-0193(1999)8: 2/3<109::AID-HBM7>3.0.CO;2-W

Dalgleish, T., Walsh, N. D., Mobbs, D., Schweizer, S., Van Harmelen, A.-L., Dunn, B., Dunn, V., Goodyer, I., & Stretton, J. (2017). Social pain and social gain in the adolescent brain: A common neural circuitry underlying both positive and negative social evaluation. *Scientific Reports*, *7* (1), 42010. <https://doi.org/10.1038/srep42010>

Diener, E., & Ryan, K. (2009). Subjective well-being: A general overview. *South African Journal of Psychology*, *39* (4), 391–406. <https://doi.org/10.1177/008124630903900402>

Dobbelaar, S., Achterberg, M., Van Drunen, L., Van Duijvenvoorde, A. C., Van IJzendoorn, M. H., & Crone, E. A. (2022). Development of social feedback processing and responses in childhood: An fMRI test-replication design in two age cohorts. *Social Cognitive and Affective Neuroscience*, nsac039. <https://doi.org/10.1093/scan/nsac039>

Dobbelaar, S., Achterberg, M., Van Duijvenvoorde, A. C., Van IJzendoorn, M. H., & Crone, E. A. (2023). Developmental patterns and individual differences in responding to social feedback: A longitudinal fMRI study from childhood to adolescence. *Developmental Cognitive Neuroscience*, *62*, 101264. <https://doi.org/10.1016/j.dcn.2023.101264>

Dodge, K. A., Lansford, J. E., Burks, V. S., Bates, J. E., Pettit, G. S., Fontaine, R., & Price, J. M. (2003). Peer rejection and social information-processing factors in the development of aggressive behavior problems in children. *Child Development*, *74* (2), 374–393. <https://doi.org/10.1111/1467-8624.7402004>

Evans, S. C., Daz, K. I., Callahan, K. P., Wolock, E. R., & Fite, P. J. (2021). Parallel trajectories of proactive and reactive aggression in middle childhood and their outcomes in early adolescence. *Research on Child and Adolescent Psychopathology*, *49* (2), 211–226. <https://doi.org/10.1007/s10802-020-00709-5>

Fair, D. A., Dosenbach, N. U., Moore, A. H., Satterthwaite, T. D., & Milham, M. P. (2021). Developmental cognitive neuroscience in the era of networks and big data: Strengths, weaknesses, opportunities, and threats. *Annual Review of Developmental Psychology*, *3* (1), 249–275. <https://doi.org/10.1146/annurev-devpsych-121318-085124>

Fite, P. J., Colder, C. R., Lochman, J. E., & Wells, K. C. (2008). Developmental trajectories of proactive and reactive aggression from fifth to ninth grade. *Journal of Clinical Child & Adolescent Psychology*, *37* (2), 412–421. <https://doi.org/10.1080/15374410801955920>

Foulkes, L., & Blakemore, S.-J. (2018). Studying individual differences in human adolescent brain development. *Nature Neuroscience*, *21* (3), 315–323. <https://doi.org/10.1038/s41593-018-0078-4>

Gallagher, H. L., & Frith, C. D. (2003). Functional imaging of “theory of mind”. *Trends in* *Cognitive Sciences*, *7* (2), 77–83. <https://doi.org/10.1016/S1364-6613(02)00025-6>

Gelman, A., & Hill, J. (2007). *Data Analysis Using Regression and Multilevel/Hierarchival Models*. Cambridge University Press.

Giancola, P. R., & Parrott, D. J. (2008). Further evidence for the validity of the Taylor Aggression Paradigm. Aggressive behavior, *34*(2), 214–229. https://doi.org/10.1002/ab.20235

Gracia-Tabuenca, Z., Moreno, M. B., Barrios, F. A., & Alcauter, S. (2021). Development of the brain functional connectome follows puberty-dependent nonlinear trajectories. *NeuroImage*, *229*, 117769. <https://doi.org/10.1016/j.neuroimage.2021.117769>

Green, K. H., Van De Groep, S., Van Der Cruijsen, R., Polak, M. G., & Crone, E. A. (2023). The Multidimensional Wellbeing in Youth Scale (MWYS): Development and psychometric properties. *Personality and Individual Differences*, *204*, 112038. <https://doi.org/10.1016/j.paid.2022.112038>

Gunther Moor, B., Van Leijenhorst, L., Rombouts, S. A., Crone, E. A., & Van Der Molen, M. W. (2010). Do you like me? Neural correlates of social evaluation and developmental trajectories. *Social Neuroscience*, *5* (5-6), 461–482. <https://doi.org/10.1080/17470910903526155>

Guyer, A. E., Choate, V. R., Pine, D. S., & Nelson, E. E. (2012). Neural circuitry underlying affective response to peer feedback in adolescence. *Social Cognitive and* *Affective Neuroscience*, *7* (1), 81–92. <https://doi.org/10.1093/scan/nsr043>

Harter, S. (1988). Developmental processes in the construction of the self. In *Integrative processes and socialization: Early to middle childhood* (pp. 45–78). Lawrence Erlbaum Associates, Inc.

Ioannidis, K., Askelund, A. D., Kievit, R. A., & Van Harmelen, A.-L. (2020). The complex neurobiology of resilient functioning after childhood maltreatment. *BMC Medicine*, *18* (1), 32. <https://doi.org/10.1186/s12916-020-1490-7>

Jorgensen, T. D., Pornprasertmanit, S., & Schoemann, A. M. (2022). semTools: Useful tools for structural equation modeling. [https://CRAN.R-project.org/package=semTools](https://CRAN.R-project.org/package%3DsemTools)

Konijn, E. A., Nije Bijvank, M., & Bushman, B. J. (2007). I wish I were a warrior: The role of wishful identification in the effects of violent video games on aggression in adolescent boys. *Developmental Psychology*, *43* (4), 1038–1044. <https://doi.org/10.1037/0012-1649.43.4.1038>

Ladd, G. W. (2006). Peer rejection, aggressive or withdrawn behavior, and psychological maladjustment from ages 5 to 12: An examination of four predictive models. *Child* *Development*, *77* (4), 822–846.

Lamblin, M., Murawski, C., Whittle, S., & Fornito, A. (2017). Social connectedness, mental health and the adolescent brain. *Neuroscience & Biobehavioral Reviews*, *80*, 57–68. <https://doi.org/10.1016/j.neubiorev.2017.05.010>

Lansford, J. E., Malone, P. S., Dodge, K. A., Pettit, G. S., & Bates, J. E. (2010). Developmental cascades of peer rejection, social information processing biases, and aggression during middle childhood. *Development and Psychopathology*, *22* (3), 593–602. <https://doi.org/10.1017/S0954579410000301>

Leary, M. R., Twenge, J. M., & Quinlivan, E. (2006). Interpersonal rejection as a determinant of anger and aggression. *Personality and Social Psychology Review*, *10* (2), 111–132. <https://doi.org/10.1207/s15327957pspr1002_2>

Lee, D., & Seo, H. (2016). Neural basis of strategic decision making. *Trends in* *Neurosciences*, *39* (1), 40–48. <https://doi.org/10.1016/j.tins.2015.11.002>

Luna, B., Garver, K. E., Urban, T. A., Lazar, N. A., & Sweeney, J. A. (2004). Maturation of cognitive processes from late childhood to adulthood. *Child Development*, *75* (5), 1357–1372. <https://doi.org/10.1111/j.1467-8624.2004.00745.x>

Luna, B., Padmanabhan, A., & O’Hearn, K. (2010). What has fMRI told us about the development of cognitive control through adolescence? *Brain and Cognition*, *72* (1), 101–113. <https://doi.org/10.1016/j.bandc.2009.08.005>

Marek, S., Tervo-Clemmens, B., Calabro, F. J., Montez, D. F., Kay, B. P., Hatoum, A. S., Donohue, M. R., Foran, W., Miller, R. L., Hendrickson, T. J., Malone, S. M., Kandala, S., Feczko, E., Miranda-Dominguez, O., Graham, A. M., Earl, E. A., Perrone, A. J., Cordova, M., Doyle, O., . . . Dosenbach, N. U. F. (2022). Reproducible brain-wide association studies require thousands of individuals. *Nature*, *603* (7902), 654–660. <https://doi.org/10.1038/s41586-022-04492-9>

Nelson, E. E., Jarcho, J. M., & Guyer, A. E. (2016). Social re-orientation and brain development: An expanded and updated view. *Developmental Cognitive* *Neuroscience*, *17*, 118–127. <https://doi.org/10.1016/j.dcn.2015.12.008>

Nelson, E. E., Leibenluft, E., McCLURE, E. B., & Pine, D. S. (2005). The social re-orientation of adolescence: A neuroscience perspective on the process and its relation to psychopathology. *Psychological Medicine*, *35* (2), 163–174. <https://doi.org/10.1017/S0033291704003915>

Nesdale, D., & Lambert, A. (2007). Effects of experimentally manipulated peer rejection on children’s negative affect, self-esteem, and maladaptive social behavior. *International Journal of Behavioral Development*, *31* (2), 115–122. <https://doi.org/10.1177/0165025407073579>

O’Shaughnessy, E. S., Berl, M. M., Moore, E. N., & Gaillard, W. D. (2008). Pediatric functional magnetic resonance imaging (fMRI): Issues and applications. *Journal of* *Child Neurology*, *23* (7), 791–801. <https://doi.org/10.1177/0883073807313047>

Prinstein, M. J., & Aikins, J. W. (2004). Cognitive moderators of the longitudinal association between peer rejection and adolescent depressive symptoms. *Journal of Abnormal Child Psychology*, *32* (2), 147–158. https://doi.org/10.1023/B:JACP.0000019767.55592.63

Prinstein, M. J., & La Greca, A. M. (2004). Childhood peer rejection and aggression as predictors of adolescent girls’ externalizing and health risk behaviors: A 6-year longitudinal study. *Journal of Consulting and Clinical Psychology*, *72* (1), 103–112. <https://doi.org/10.1037/0022-006X.72.1.103>

Quarmley, M., Feldman, J., Grossman, H., Clarkson, T., Moyer, A., & Jarcho, J. M. (2022). Testing effects of social rejection on aggressive and prosocial behavior: A meta-analysis. Aggressive behavior, 48(6), 529–545. https://doi.org/10.1002/ab.22026

R Core Team. (2022). R: A language and environment for statistical computing.

Riva, P., Romero Lauro, L. J., DeWall, C. N., Chester, D. S., & Bushman, B. J. (2015). Reducing aggressive responses to social exclusion using transcranial direct current stimulation. *Social Cognitive and Affective Neuroscience*, *10* (3), 352–356. <https://doi.org/10.1093/scan/nsu053>

Rosseel, Y. (2012). Lavaan: An R package for structural equation modeling. *Journal of* *statistical Software*, *48* (2). <https://doi.org/10.18637/jss.v048.i02>

Rubin, D. B. (Ed.). (1987). *Multiple Imputation for Nonresponse in Surveys*. John Wiley & Sons, Inc. <https://doi.org/10.1002/9780470316696>

Rudolph, K. D., Davis, M. M., Skymba, H. V., Modi, H. H., & Telzer, E. H. (2021). Social experience calibrates neural sensitivity to social feedback during adolescence: A functional connectivity approach. *Developmental Cognitive Neuroscience*, *47*, 100903. <https://doi.org/10.1016/j.dcn.2020.100903>

Schriber, R. A., & Guyer, A. E. (2016). Adolescent neurobiological susceptibility to social context. *Developmental Cognitive Neuroscience*, *19*, 1–18. <https://doi.org/10.1016/j.dcn.2015.12.009>

Somerville, L. H., & Casey, B. (2010). Developmental neurobiology of cognitive control and motivational systems. *Current Opinion in Neurobiology*, *20* (2), 236–241. <https://doi.org/10.1016/j.conb.2010.01.006>

Somerville, L. H., Heatherton, T. F., & Kelley, W. M. (2006). Anterior cingulate cortex responds differentially to expectancy violation and social rejection. *Nature* *Neuroscience*, *9* (8), 1007–1008. <https://doi.org/10.1038/nn1728>

Steinberg, L. (2008). A social neuroscience perspective on adolescent risk-taking. *Developmental Review*, *28* (1), 78–106. <https://doi.org/10.1016/j.dr.2007.08.002> Steinberg, L., & Morris, A. S. (2001). Adolescent development. *Annual Review of* *Psychology*, *52* (1), 83–110. <https://doi.org/10.1146/annurev.psych.52.1.83>

Telzer, E. H., McCormick, E. M., Peters, S., Cosme, D., Pfeifer, J. H., & Van Duijvenvoorde, A. C. (2018). Methodological considerations for developmental longitudinal fMRI research. *Developmental Cognitive Neuroscience*, *33*, 149–160. <https://doi.org/10.1016/j.dcn.2018.02.004>

Vahedi, S. (2010). World Health Organization Quality-of-Life Scale (WHOQOL-BREF): Analyses of their item response theory properties based on the graded responses model. *Iranian journal of psychiatry*, *5* (4), 140–153.

Van de Groep, I. H., Bos, M. G., Jansen, L. M., Achterberg, M., Popma, A., & Crone, E. A. (2021). Overlapping and distinct neural correlates of self-evaluations and self-regulation from the perspective of self and others. *Neuropsychologia*, *161*, 108000. <https://doi.org/10.1016/j.neuropsychologia.2021.108000>

Van de Groep, I. H., Bos, M. G., Jansen, L. M., Kocevska, D., Bexkens, A., Cohn, M., Van Domburgh, L., Popma, A., & Crone, E. A. (2022). Resisting aggression in social contexts: The influence of life-course persistent antisocial behavior on behavioral and neural responses to social feedback. *NeuroImage: Clinical*, *34*, 102973. <https://doi.org/10.1016/j.nicl.2022.102973>

Van der Meulen, M., Dobbelaar, S., Van Drunen, L., Heunis, S., Van IJzendoorn, M. H., Blankenstein, N. E., & Crone, E. A. (2023). Transitioning from childhood into adolescence: A comprehensive longitudinal behavioral and neuroimaging study on prosocial behavior and social inclusion. *NeuroImage*, *284*, 120445. <https://doi.org/10.1016/j.neuroimage.2023.120445>

Van Harmelen, A.-L., Blakemore, S. J., Goodyer, I. M., & Kievit, R. A. (2021). The interplay between adolescent friendship quality and resilient functioning following childhood and adolescent adversity. *Adversity and Resilience Science*, *2* (1), 37–50. <https://doi.org/10.1007/s42844-020-00027-1>

Van Harmelen, A.-L., Kievit, R. A., Ioannidis, K., Neufeld, S., Jones, P. B., Bullmore, E., Dolan, R., The NSPN Consortium, Fonagy, P., & Goodyer, I. (2017). Adolescent friendships predict later resilient functioning across psychosocial domains in a healthy community cohort. *Psychological Medicine*, *47* (13), 2312–2322. <https://doi.org/10.1017/S0033291717000836>

Vijayakumar, N., Pfeifer, J. H., Flournoy, J. C., Hernandez, L. M., & Dapretto, M. (2019). Affective reactivity during adolescence: Associations with age, puberty and testosterone. *Cortex*, *117*, 336–350. <https://doi.org/10.1016/j.cortex.2019.04.024>

Welsh, M., & Peterson, E. (2014). Issues in the conceptualization and assessment of hot executive functions in childhood. *Journal of the International Neuropsychological* *Society*, *20* (2), 152–156. <https://doi.org/10.1017/S1355617713001379>

Wichstraum, L. (1995). Harter’s Self-Perception Profile for adolescents: Reliability, validity, and evaluation of the question format. *Journal of Personality Assessment*, *65* (1), 100–116. <https://doi.org/10.1207/s15327752jpa6501_8>

Zelazo, P. D., & Carlson, S. M. (2012). Hot and cool executive function in childhood and adolescence: Development and plasticity. *Child Development Perspectives*, n/a–n/a. <https://doi.org/10.1111/j.1750-8606.2012.00246.x>

Zhu J, Kusa TO, Chan YM. (2018). Genetics of pubertal timing. *Curr Opin Pediatr,*

*30*(4):532-540. doi: 10.1097/MOP.0000000000000642.

# Table 1

*Overview of participant demographics, age ranges, and exclusions per measurement wave of the Leiden Consortium on Individual Development.*

|  | Wave 1 | Wave 3 | Wave 5 | Wave 6 |
| --- | --- | --- | --- | --- |
| *N* | 512 | 456 | 336 | 294 |
| Mean age (years) | 7.94 | 9.98 | 12.38 | 13.34 |
| Range age (years) | 7.02-9.68 | 8.97-11.67 | 11.15-14.11 | 11.98-15.10 |
| Female (percentage) | 51% | 52% | 53% | 54% |
| Right-handed (percentage) | 87% | 87% | 85% | 87% |
| *n*_MRI completed_a | 485 | 408 | 236 | - |
| MRI exclusions: |  |  |  |  |
| *n*_anomalous findings_ | 4 | 0 | 0 | - |
| *n*_excessive head motion_ | 89 | 38 | 13 | - |
| *n*_data export/processing error_ | 9 | 5 | 1 | - |
| *n*_MRI inclusion_ | 383 | 365 | 222 | - |

a Not all participants in the sample completed an MRI scans due to anxiety, MRI contra indications (e.g., braces), no parental consent, or technical problems.

# Table 2

*Point estimates and 95% credible intervals for intraclass-correlations for behavioral and neural responses across wave-, individual-, and family levels. The asterisk * denotes that the posterior probability of the estimate being greater than zero exceeds 95%.*

|  | Wavea | Individual | Family |
| --- | --- | --- | --- |
| Aggression (noise) | 0.29 [0.27-0.31]* | 0.03 [0.01-0.06]* | 0.03 [0.01-0.05]* |
| AI | - | 0.01 [0.00-0.03]*b | 0.01 [0.00-0.02]*b |
| MPFC | - | 0.02 [0.00-0.07]*b | 0.04 [0.00-0.09]*b |
| DLPFC | - | 0.01 [0.00-0.05]*b | 0.01 [0.00-0.03]*b |

a Neural responses were not analyzed on a trial-by-trial basis as part of the multilevel hence. Hence, the wave-level is the “lowest” level in the neural data, and the grouping structure only exists as the individual- and family-levels.

b Due to rounding, these credible intervals contain zero. However, results show that the posterior probability of the estimate being greater than zero exceeds 95%.

# Table 3

*95% credible intervals for the fixed effects (FEs) and the standard deviations of the random effects (REs) of the growth components. REs exist at both the individual-level (i.e., within families) and the family-level (i.e., between families). Results are shown for development in behavioral aggression, and neural responses in the anterior insula, media prefrontal cortex, and dorsolateral prefrontal cortex. The asterisk* ∗ *denotes credible intervals not containing zero.*

|  | FE | *SD*(RE) individual-level | *SD*(RE) family-level |
| --- | --- | --- | --- |
| Aggression (noise) |  |  |  |
| Intercept | [1.31, 1.49]∗ | [0.03, 0.42]∗ | [0.15, 0.43]∗ |
| Linear slope | [-0.05, 0.02] | [0.00, 0.11] | [0.01, 0.16]∗ |
| Quadratic slope  AI | [-0.06, -0.03]∗ | [0.00, 0.03] | [0.00, 0.05] |
| Intercept | [0.42, 1.03]∗ | [0.02, 1.01]∗ | [0.01, 0.81]∗ |
| Linear slope | [-0.29, -0.02]∗ | [0.03, 0.66]∗ | [0.01, 0.35]∗ |
| Quadratic slope  MPFC | [-0.01, 0.13] | [0.00, 0.17] | [0.00, 0.14] |
| Intercept | [0.52, 1.20]∗ | [0.03, 1.37]∗ | [0.13, 1.62]∗ |
| Linear slope | [-0.17, 0.10] | [0.01, 0.56]∗ | [0.00, 0.31] |
| Quadratic slope  DLPFC | [-0.01, 0.13] | [0.00, 0.23] | [0.00, 0.27] |
| Intercept | [-0.65, -0.09]∗ | [0.02, 0.97]∗ | [0.01, 0.84]∗ |
| Linear slope | [-0.14, 0.11] | [0.02, 0.53]∗ | [0.01, 0.43]∗ |
| Quadratic slope | [-0.02, 0.11] | [0.00, 0.17] | [0.00, 0.16] |

# Table 4

*Parameter estimates of the measurement model of social well-being. The factor loading of the first indicator was set to one for scaling. AWP = ten items from the Adolescent Wellbeing Paradigm; WHO = ten items from the World Health Organization Quality of Life Scale; SC = Social Competence subscale from Harter’s Self-Perception Profile for Adolescents; CF = Close Friendships subscale of Harter’s Self-Perception Profile for Adolescents; GS = Global Self-Worth subscale of Harter’s Self-Perception Profile for Adolescents.*

| Parameter | Est. | SE | 95% CI |
| --- | --- | --- | --- |
| Factor loadings: |  |  |  |
| *λ_AWP_* | 1 | - | - |
| *λ_WHO_* | 0.996 | 0.057 | [0.884, 1.108] |
| *λ_SC_* | 0.766 | 0.096 | [0.578, 0.954] |
| *λ_CF_* | 0.628 | 0.092 | [0.448, 0.808] |
| *λ_GS_*  Unique variances: | 1.161 | 0.099 | [0.967, 1.355] |
| *θ_AWP_* | 0.051 | 0.007 | [0.037, 0.065] |
| *θ_WHO_* | 0.019 | 0.006 | [0.007, 0.031] |
| *θ_SC_* | 0.299 | 0.026 | [0.248, 0.350] |
| *θ_CF_* | 0.281 | 0.024 | [0.233, 0.328] |
| *θ_GS_*  Common variance: | 0.275 | 0.025 | [0.226, 0.324] |
| *ψ* | 0.141 | 0.017 | [0.108, 0.174] |


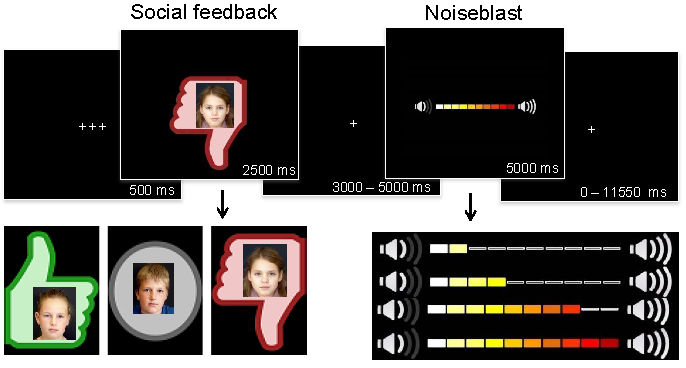


# Figure 1

*Social Network Aggression Task. After the participants viewed positive, neutral or negative social feedback on their personal profile, participants got the opportunity to blast a loud noise towards the peer, which was taken as a proxy for behavioral aggression following social* ***feedback****.*


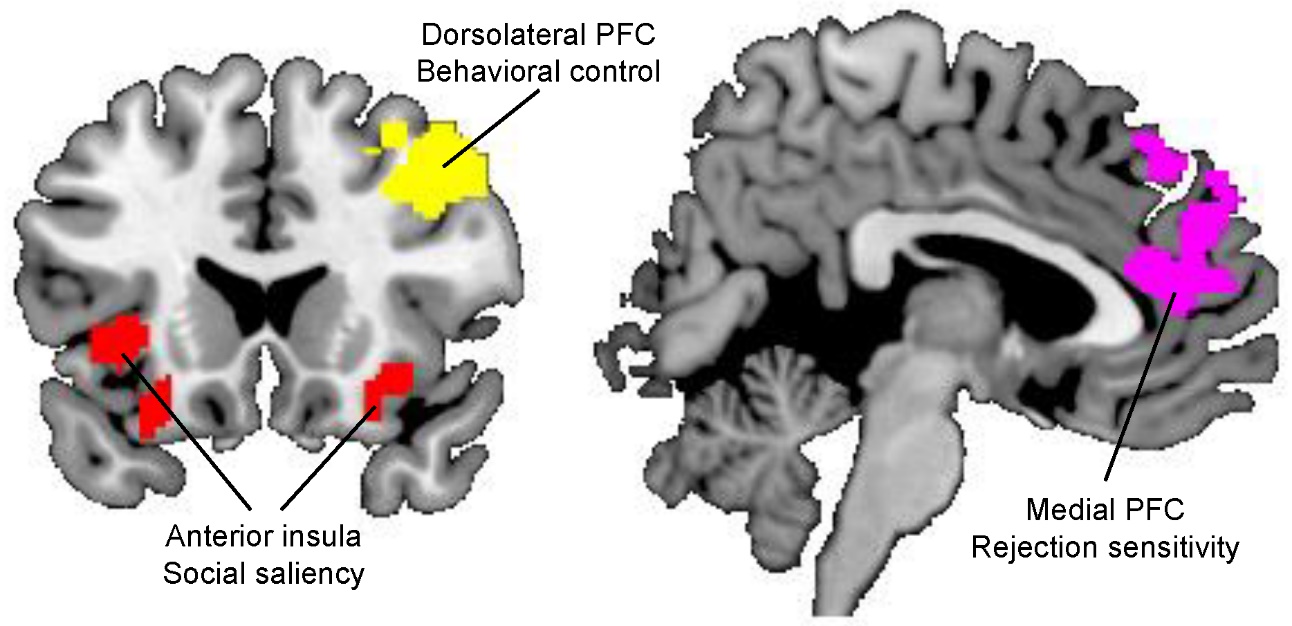


**Figure 2**

*Regions of interest (ROIs) for the anterior insula (AI), the medial prefrontal cortex (MPFC), and the right dorsolateral prefrontal cortex (DLPFC). ROIs are openly accessible through* [*https://osf.io/byn7r/files/*](https://osf.io/byn7r/files/) *(in .png, .nii, and .mat files).*


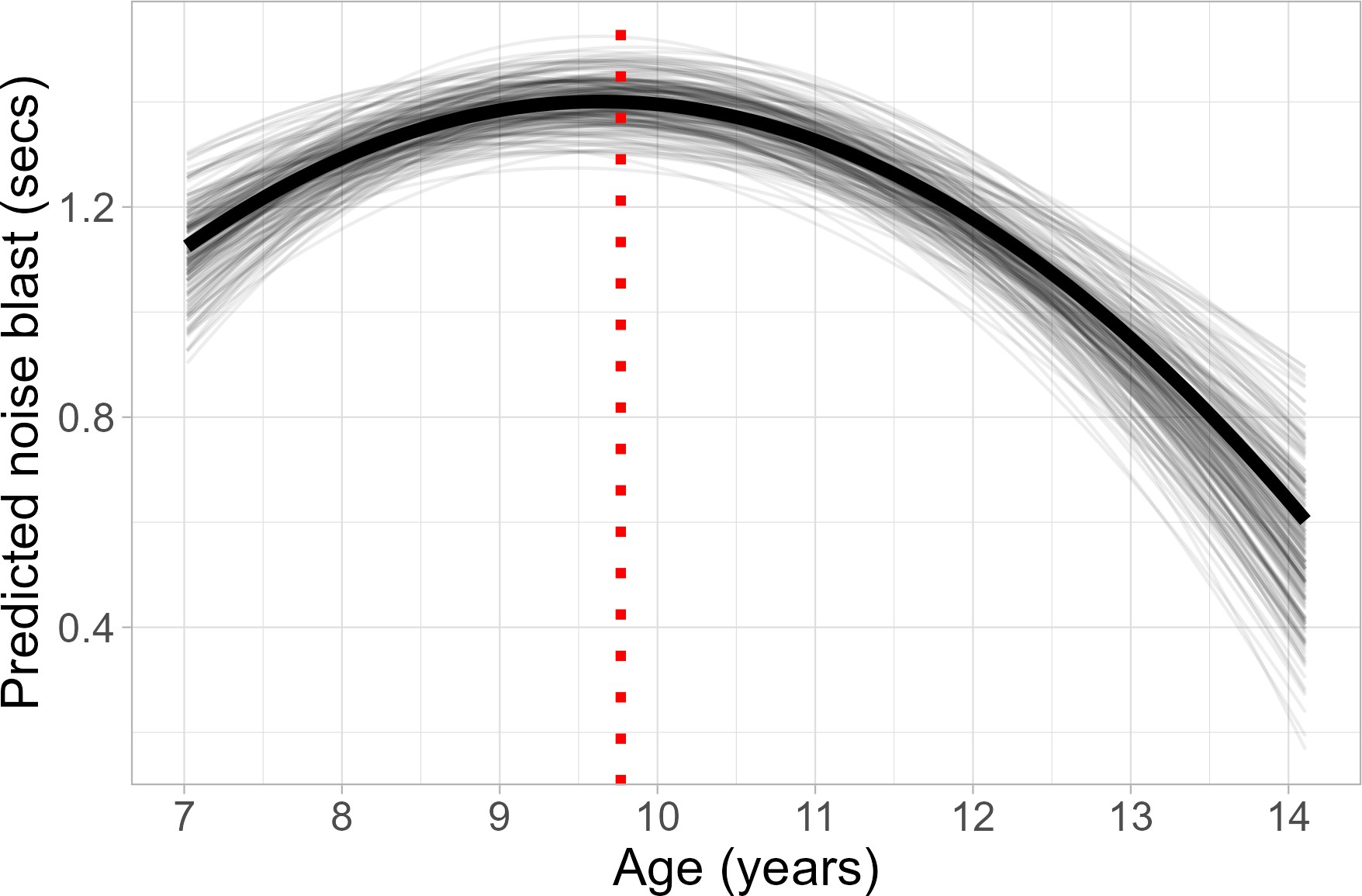

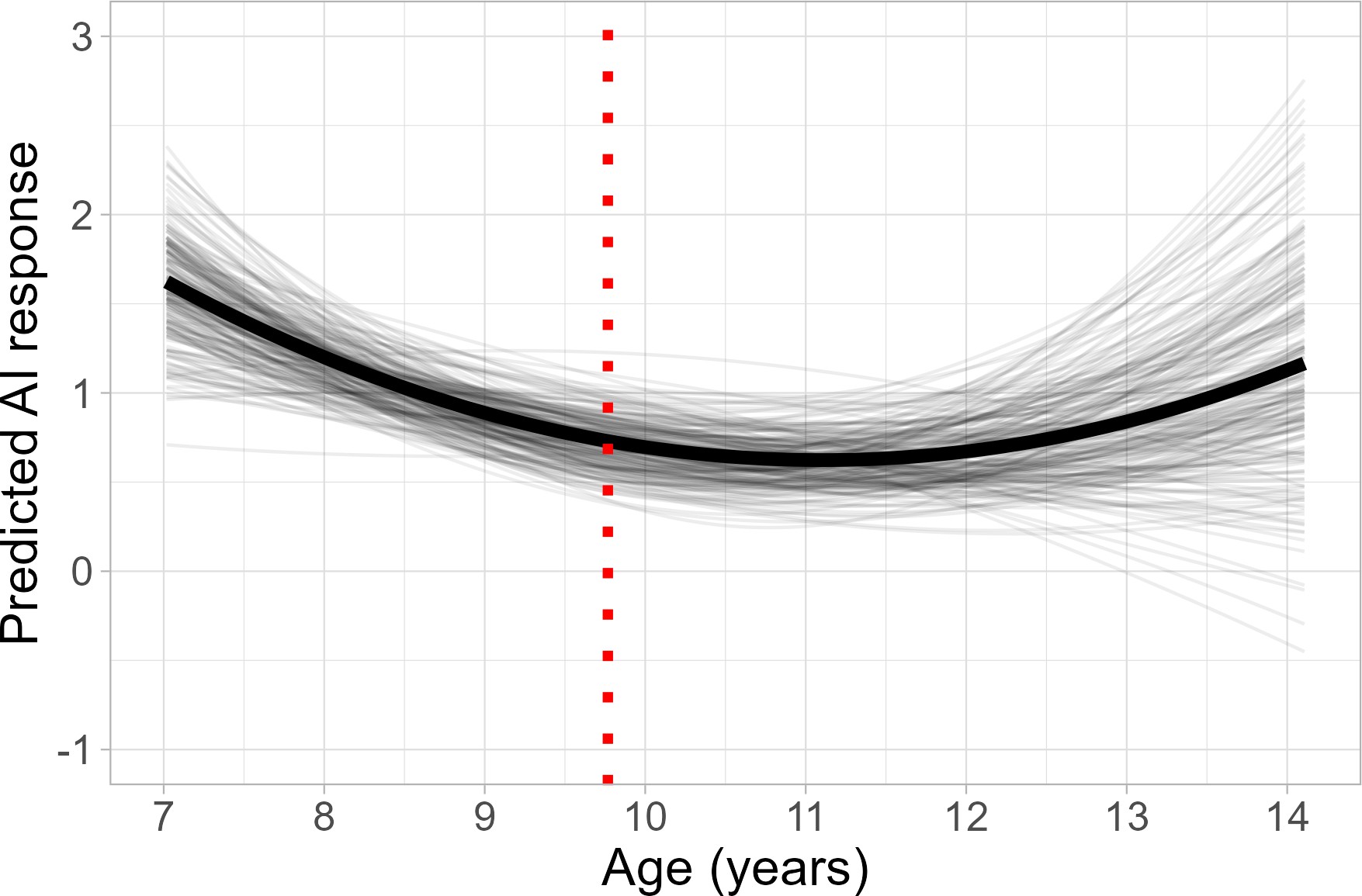


(a) *Behavioral aggressive responses.* (b) *Neural (AI) responses.*


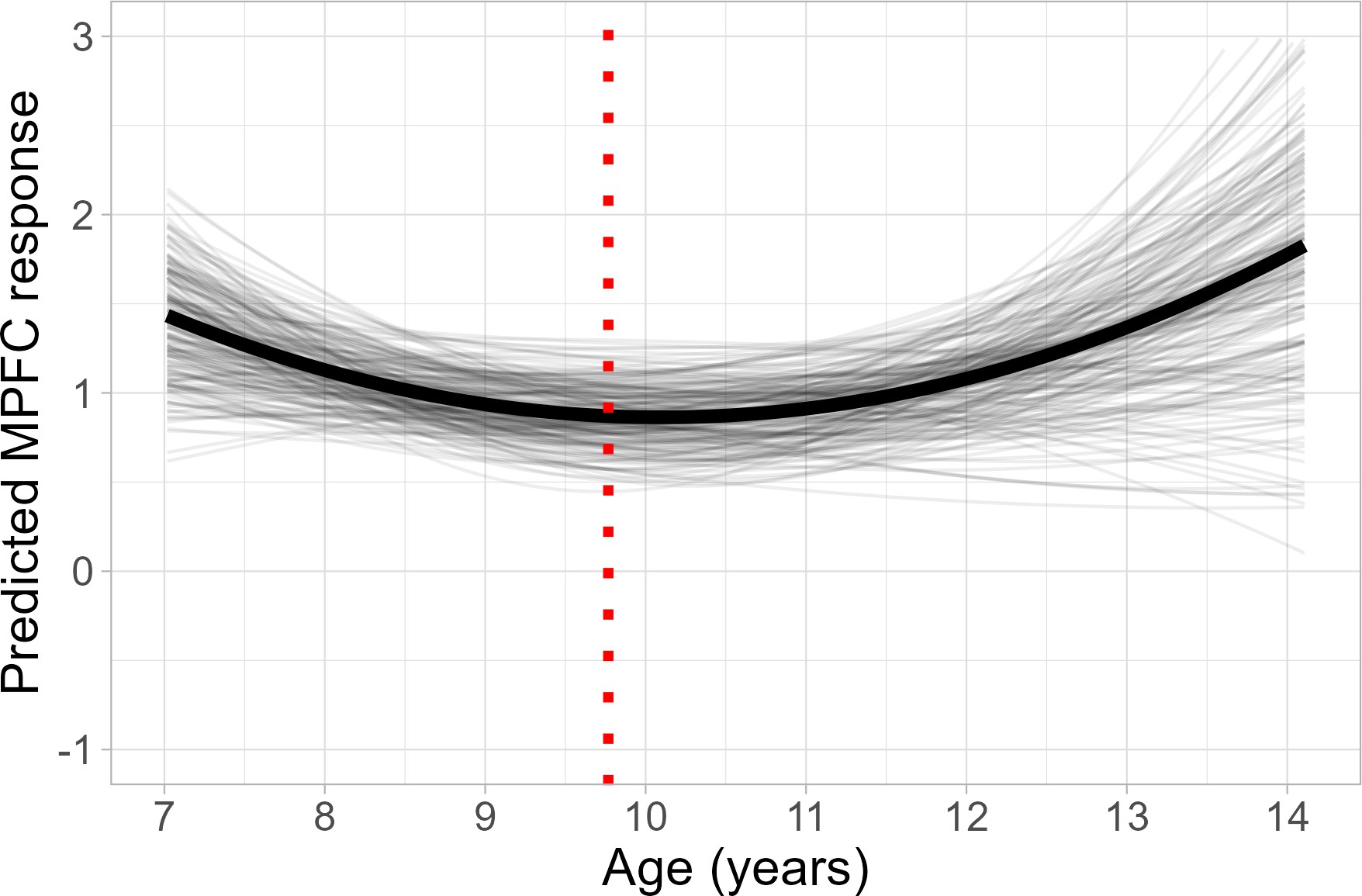

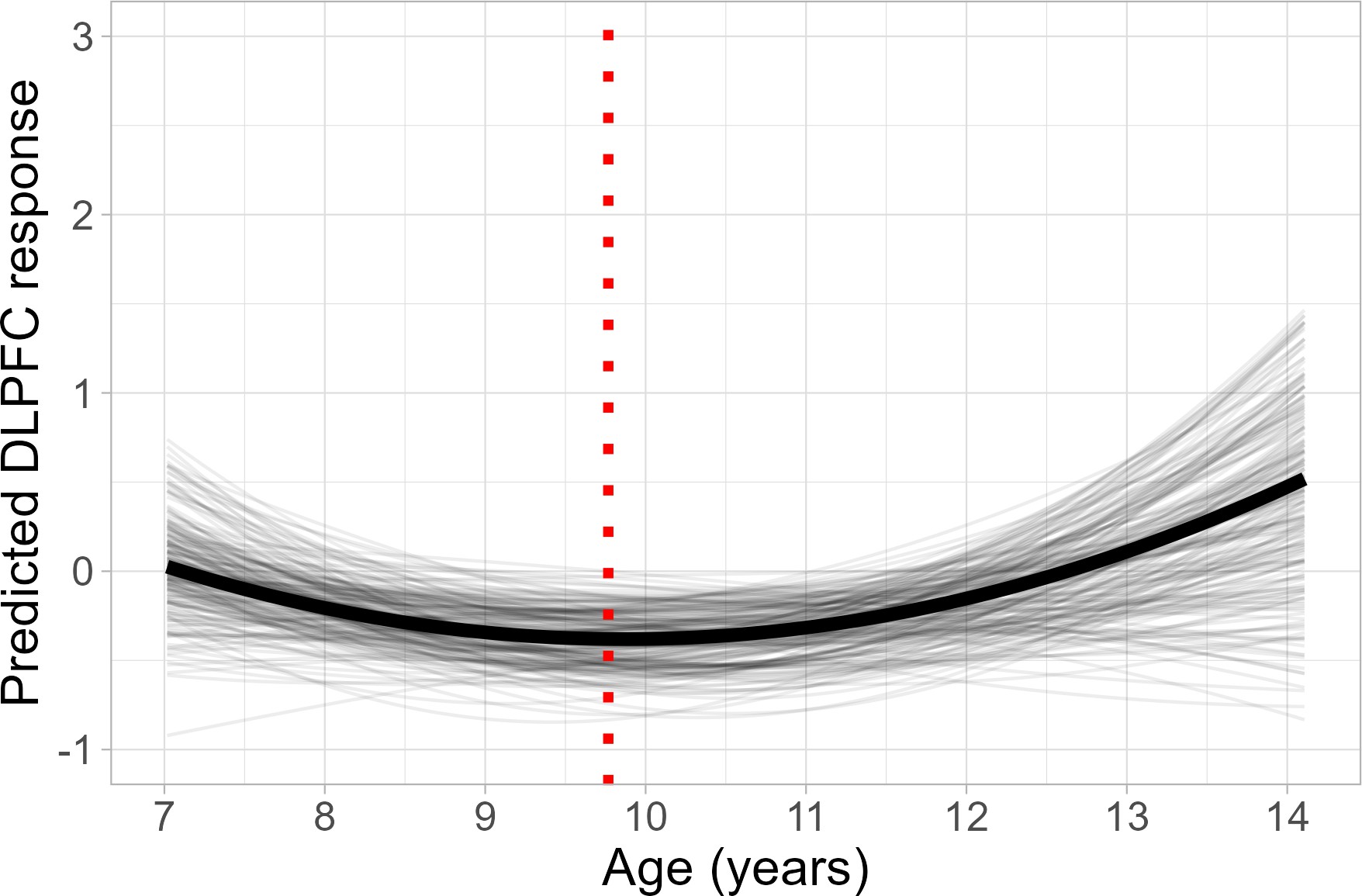


(c) *Neural (MPFC) responses.* (d) *Neural (DLPFC) responses.*

# Figure 3

*Model-predicted development in behavioral and neural responses to negative versus neutral social feedback. The bold black line represents the predicted (based on the fixed effects) average development across adolescence. The gray lines represent uncertainty around this prediction, based on draws from the posterior distribution for the fixed effects. The vertical dotted (red) line represents the mean age of approximately 9 years and 9 months.*

1. Note that there is some overlap in the sample used for these studies. Achterberg et al. (2018) examined the first waves of the L-CID middle childhood cohort (MCC). Dobbelaar et al. (2022) examined the first wave of the L-CID MCC and replicated findings in the fifth wave of the L-CID early childhood cohort (ECC). Dobbelaar et al. (2023) examined the first, third, and fifth wave of the L-CID MCC sample. Achterberg et al. (2020) examined neural responses to rejection in the first and third wave of the L-CID MCC sample; the current study extends this work by additionally analyzing the fifth wave of L-CID MCC sample. [↑](#footnote-ref-1)
2. Because the variable time was grand mean centered before use in the multilevel model, the growth components represent development at the mean age of participants. This was done to prevent issues with multicollinearity of the linear and quadratic growth components in the model. [↑](#footnote-ref-2)
3. In contrast to the preregistration, we did not use the software package Mplus. This decision was made for convenience as R packages are freely and openly available. [↑](#footnote-ref-3)
